# PTSD in jewish holocaust survivors’ as a risk factor in the development of mental health conditions in their offspring

**DOI:** 10.1192/j.eurpsy.2021.1200

**Published:** 2021-08-13

**Authors:** S. Cooper

**Affiliations:** Pyrland Wand, Somerset Foundation Trust, Cheddon, Taunton, United Kingdom

**Keywords:** Holocaust, post-traumatic stress disorder, mental health, Offspring

## Abstract

**Introduction:**

Transgenerational transmission of trauma (TTT) describes the residual ‘presence of the past’ through generations. This phenomenon has an established evidence base with Holocaust survivors (HS) and their offspring, who are hypothesised to be at a greater risk of psychiatric conditions. This advanced literature review explores the relationship between Post Traumatic Stress Disorder (PTSD) in survivors and mental health conditions (MHC) in survivor’s offspring.

**Objectives:**

The objective is to review the literature, looking for evidence of TTT and exploring the mechanisms of action of such phenomenon.

**Methods:**

An advanced search was performed in three databases; Medline, Ovid PsycInfo and the Yehuda Schwarzbaum Online library using the following search terms; (Post Traumatic Stress Disorder OR PTSD) AND (Holocaust OR Shoah) AND (Offspring OR Children)’. 190 articles were identified and a following 163 were excluded. 26 studies were reviewed.

**Results:**

Parental PTSD is circumstantially influential in parenting and attachment quality. Unfavourable attachments in offspring are associated with psychiatric conditions. Furthermore, poor health behaviour can be transmitted; for example, poor diet is an independent risk factor for depression. Psychopathology may pass intergenerationally; parental PTSD increases the risk of developing PTSD in response to one’s trauma. Parental PTSD can also result in impaired cortisol function and epigenetic changes.

**Conclusions:**

PTSD in HS is an important risk factor for development of MHC in offspring. However, this does not mean all offspring develop MHC. The variability in offspring proneness to psychiatric conditions may reflect specific vulnerabilities. Further research is pertinent for an understanding of TTT. The poster will discuss clinical value.

